# Supplementary material for: Colonization of the human gut by bovine bacteria present in Parmesan cheese
Source: Nat Commun. 2019 Mar 20;10:1286. doi: 10.1038/s41467-019-09303-w (PMC6426854; doi:10.1038/s41467-019-09303-w)
Supplement: Supplementary file 1 — Supplementary Information [file 41467_2019_9303_MOESM1_ESM.pdf]

Supplementary Information

Colonization of the human gut by bovine bacteria present in Parmesan cheese

Key words: bifidobacteria, metagenomics, diet, cheese, food microorganisms, RNAseq

Christian Milani[1], Sabrina Duranti[1], Stefania Napoli[2], Giulia Alessandri[3], Leonardo Mancabelli[2], Rosaria Anzalone[2], Giulia Longhi[2], Alice Viappiani[2], Marta Mangifesta[1,2], Gabriele Andrea Lugli[1], Sergio Bernasconi[4], Maria Cristina Ossiprandi[5], Douwe van Sinderen[5,6,7], Marco Ventura[1,4,*] and Francesca Turroni[1,4,*]

Laboratory of Probiogenomics, Department of Chemistry, Life Sciences and Environmental Sustainability, University of Parma, Italy[1]; GenProbio srl, Parma, Italy[2]; Department of Veterinary Science, University of Parma, Italy[3]; Microbiome Research Hub, University of Parma, Parma, Italy[4]; Department of Veterinary Medical Science, University of Parma, Parma, Italy[5]; APC Microbiome Ireland[6] and School of Microbiology[7], University College Cork, Cork, Ireland.

*These authors contributed equally to this work.

Corresponding author: Prof. Marco Ventura, Department of Chemistry, Life Sciences and Environmental Sustainability, University of Parma, Parco Area delle Scienze 11a, 43124 Parma, Italy. Phone: +39-0521-905666. Fax: +39-521-905604. E-mail: marco.ventura@unipr.it

Francesca Turroni Department of Life Sciences, University of Parma, Parco Area delle Scienze 11a, 43124 Parma, Italy. Phone: +39-0521-905666. Fax: +39-521-905604. E-mail: francesca.turroni@unipr.it

Supplementary Table 1: Detailed information regarding samples included in this study.

Samples name	Husbandry	Samples type	Breed	Feed	Housing
**P1_CF_1**	Parma 1	faecal	Friesian cow	Hay	Tied up
**P1_CF_2**	Parma 1	faecal	Friesian cow	Hay	Tied up
**P1_CF_3**	Parma 1	faecal	Friesian cow	Hay	Tied up
**P1_CF_4**	Parma 1	faecal	Friesian cow	Hay	Tied up
**P1_CF_5**	Parma 1	faecal	Friesian cow	Hay	Tied up
**P1_CF_6**	Parma 1	faecal	Friesian cow	Hay	Tied up
**P1_CF_7**	Parma 1	faecal	Friesian cow	Hay	Tied up
**P1_CF_8**	Parma 1	faecal	Friesian cow	Hay	Tied up
**P1_CF_9**	Parma 1	faecal	Friesian cow	Hay	Tied up
**P1_CF_10**	Parma 1	faecal	Friesian cow	Hay	Tied up
**P1_MIL_1**	Parma 1	Milk	Friesian cow	Hay	Tied up
**P1_MIL_2**	Parma 1	Milk	Friesian cow	Hay	Tied up
**P1_MIL_3**	Parma 1	Milk	Friesian cow	Hay	Tied up
**P1_MIL_4**	Parma 1	Milk	Friesian cow	Hay	Tied up
**P1_MIL_5**	Parma 1	Milk	Friesian cow	Hay	Tied up
**P1_MIL_6**	Parma 1	Milk	Friesian cow	Hay	Tied up
**P1_MIL_7**	Parma 1	Milk	Friesian cow	Hay	Tied up
**P1_MIL_8**	Parma 1	Milk	Friesian cow	Hay	Tied up
**P1_MIL_9**	Parma 1	Milk	Friesian cow	Hay	Tied up
**P1_MIL_10**	Parma 1	Milk	Friesian cow	Hay	Tied up
**P1_LIT_1**	Parma 1	Litter			
**P1_LIT_2**	Parma 1	Litter			
**P1_LIT_3**	Parma 1	Litter			
**P1_LIT_4**	Parma 1	Litter			
**P1_LIT_5**	Parma 1	Litter			
**P1_LIT_6**	Parma 1	Litter			
**P1_LIT_7**	Parma 1	Litter			
**P1_LIT_8**	Parma 1	Litter			
**P1_LIT_9**	Parma 1	Litter			
**P1_LIT_10**	Parma 1	Litter			
**P1_PC_1**	Parma 1	Parmesan Cheese	Friesian cow	Hay	Tied up
**P1_PC_2**	Parma 1	Parmesan Cheese	Friesian cow	Hay	Tied up
**P1_PC_3**	Parma 1	Parmesan Cheese	Friesian cow	Hay	Tied up
**P2_CF_1**	Parma 2	faecal	Friesian cow	Hay	Unbound
**P2_CF_2**	Parma 2	faecal	Friesian cow	Hay	Unbound
**P2_CF_3**	Parma 2	faecal	Friesian cow	Hay	Unbound
**P2_CF_4**	Parma 2	faecal	Friesian cow	Hay	Unbound
**P2_CF_5**	Parma 2	faecal	Friesian cow	Hay	Unbound
**P2_CF_6**	Parma 2	faecal	Friesian cow	Hay	Unbound
**P2_CF_7**	Parma 2	faecal	Friesian cow	Hay	Unbound
**P2_CF_8**	Parma 2	faecal	Friesian cow	Hay	Unbound

**P2_CF_9**	Parma 2	faecal	Friesian cow	Hay	Unbound
**P2_CF_10**	Parma 2	faecal	Friesian cow	Hay	Unbound
**P2_MIL_1**	Parma 2	Milk	Friesian cow	Hay	Unbound
**P2_MIL_2**	Parma 2	Milk	Friesian cow	Hay	Unbound
**P2_MIL_3**	Parma 2	Milk	Friesian cow	Hay	Unbound
**P2_MIL_4**	Parma 2	Milk	Friesian cow	Hay	Unbound
**P2_MIL_5**	Parma 2	Milk	Friesian cow	Hay	Unbound
**P2_MIL_6**	Parma 2	Milk	Friesian cow	Hay	Unbound
**P2_MIL_7**	Parma 2	Milk	Friesian cow	Hay	Unbound
**P2_MIL_8**	Parma 2	Milk	Friesian cow	Hay	Unbound
**P2_MIL_9**	Parma 2	Milk	Friesian cow	Hay	Unbound
**P2_MIL_10**	Parma 2	Milk	Friesian cow	Hay	Unbound
**P2_LIT_1**	Parma 2	Litter			
**P2_LIT_2**	Parma 2	Litter			
**P2_LIT_3**	Parma 2	Litter			
**P2_LIT_4**	Parma 2	Litter			
**P2_LIT_5**	Parma 2	Litter			
**P2_LIT_6**	Parma 2	Litter			
**P2_LIT_7**	Parma 2	Litter			
**P2_LIT_8**	Parma 2	Litter			
**P2_LIT_9**	Parma 2	Litter			
**P2_LIT_10**	Parma 2	Litter			
**P2_PC_1**	Parma 2	Parmesan Cheese	Friesian cow	Hay	Unbound
**P2_PC_2**	Parma 2	Parmesan Cheese	Friesian cow	Hay	Unbound
**P2_PC_3**	Parma 2	Parmesan Cheese	Friesian cow	Hay	Unbound
**P3_CF_1**	Parma 3	faecal	Friesian cow	Hay	Unbound
**P3_CF_2**	Parma 3	faecal	Friesian cow	Hay	Unbound
**P3_CF_3**	Parma 3	faecal	Friesian cow	Hay	Unbound
**P3_CF_4**	Parma 3	faecal	Friesian cow	Hay	Unbound
**P3_CF_5**	Parma 3	faecal	Friesian cow	Hay	Unbound
**P3_CF_6**	Parma 3	faecal	Friesian cow	Hay	Unbound
**P3_CF_7**	Parma 3	faecal	Friesian cow	Hay	Unbound
**P3_CF_8**	Parma 3	faecal	Friesian cow	Hay	Unbound
**P3_CF_9**	Parma 3	faecal	Friesian cow	Hay	Unbound
**P3_CF_10**	Parma 3	faecal	Friesian cow	Hay	Unbound
**P3_MIL_1**	Parma 3	Milk	Friesian cow	Hay	Unbound
**P3_MIL_2**	Parma 3	Milk	Friesian cow	Hay	Unbound
**P3_MIL_3**	Parma 3	Milk	Friesian cow	Hay	Unbound
**P3_MIL_4**	Parma 3	Milk	Friesian cow	Hay	Unbound
**P3_MIL_5**	Parma 3	Milk	Friesian cow	Hay	Unbound
**P3_MIL_6**	Parma 3	Milk	Friesian cow	Hay	Unbound
**P3_MIL_7**	Parma 3	Milk	Friesian cow	Hay	Unbound
**P3_MIL_8**	Parma 3	Milk	Friesian cow	Hay	Unbound
**P3_MIL_9**	Parma 3	Milk	Friesian cow	Hay	Unbound

**P3_MIL_10**	Parma 3	Milk	Friesian cow	Hay	Unbound
**P3_LIT_1**	Parma 3	Litter			
**P3_LIT_2**	Parma 3	Litter			
**P3_LIT_3**	Parma 3	Litter			
**P3_LIT_4**	Parma 3	Litter			
**P3_LIT_5**	Parma 3	Litter			
**P3_LIT_6**	Parma 3	Litter			
**P3_LIT_7**	Parma 3	Litter			
**P3_LIT_8**	Parma 3	Litter			
**P3_LIT_9**	Parma 3	Litter			
**P3_LIT_10**	Parma 3	Litter			
**P3_PC_1**	Parma 3	Parmesan Cheese	Friesian cow	Hay	Unbound
**P3_PC_2**	Parma 3	Parmesan Cheese	Friesian cow	Hay	Unbound
**P3_PC_3**	Parma 3	Parmesan Cheese	Friesian cow	Hay	Unbound
**RE1_CF_1**	Reggio Emilia 1	faecal	Friesian cow	Hay	Tied up
**RE1_CF_2**	Reggio Emilia 1	faecal	Friesian cow	Hay	Tied up
**RE1_CF_3**	Reggio Emilia 1	faecal	Friesian cow	Hay	Tied up
**RE1_CF_4**	Reggio Emilia 1	faecal	Friesian cow	Hay	Tied up
**RE1_CF_5**	Reggio Emilia 1	faecal	Friesian cow	Hay	Tied up
**RE1_CF_6**	Reggio Emilia 1	faecal	Friesian cow	Hay	Tied up
**RE1_CF_7**	Reggio Emilia 1	faecal	Friesian cow	Hay	Tied up
**RE1_CF_8**	Reggio Emilia 1	faecal	Friesian cow	Hay	Tied up
**RE1_CF_9**	Reggio Emilia 1	faecal	Friesian cow	Hay	Tied up
**RE1_CF_10**	Reggio Emilia 1	faecal	Friesian cow	Hay	Tied up
**RE1_MIL_1**	Reggio Emilia 1	Milk	Friesian cow	Hay	Tied up
**RE1_MIL_2**	Reggio Emilia 1	Milk	Friesian cow	Hay	Tied up
**RE1_MIL_3**	Reggio Emilia 1	Milk	Friesian cow	Hay	Tied up
**RE1_MIL_4**	Reggio Emilia 1	Milk	Friesian cow	Hay	Tied up
**RE1_MIL_5**	Reggio Emilia 1	Milk	Friesian cow	Hay	Tied up
**RE1_MIL_6**	Reggio Emilia 1	Milk	Friesian cow	Hay	Tied up
**RE1_MIL_7**	Reggio Emilia 1	Milk	Friesian cow	Hay	Tied up
**RE1_MIL_8**	Reggio Emilia 1	Milk	Friesian cow	Hay	Tied up
**RE1_MIL_9**	Reggio Emilia 1	Milk	Friesian cow	Hay	Tied up
**RE1_MIL_10**	Reggio Emilia 1	Milk	Friesian cow	Hay	Tied up
**RE1_LIT_1**	Reggio Emilia 1	Litter			
**RE1_LIT_2**	Reggio Emilia 1	Litter			
**RE1_LIT_3**	Reggio Emilia 1	Litter			
**RE1_LIT_4**	Reggio Emilia 1	Litter			
**RE1_LIT_5**	Reggio Emilia 1	Litter			
**RE1_LIT_6**	Reggio Emilia 1	Litter			
**RE1_LIT_7**	Reggio Emilia 1	Litter			
**RE1_LIT_8**	Reggio Emilia 1	Litter			
**RE1_LIT_9**	Reggio Emilia 1	Litter			
**RE1_LIT_10**	Reggio Emilia 1	Litter			

**RE1_PC_1**	Reggio Emilia 1	Parmesan Cheese	Friesian cow	Hay	Tied up
**RE1_PC_2**	Reggio Emilia 1	Parmesan Cheese	Friesian cow	Hay	Tied up
**RE1_PC_3**	Reggio Emilia 1	Parmesan Cheese	Friesian cow	Hay	Tied up
**RE2_CF_1**	Reggio Emilia 2	faecal	Red cow	Hay	Unbound
**RE2_CF_2**	Reggio Emilia 2	faecal	Red cow	Hay	Unbound
**RE2_CF_3**	Reggio Emilia 2	faecal	Red cow	Hay	Unbound
**RE2_CF_4**	Reggio Emilia 2	faecal	Red cow	Hay	Unbound
**RE2_CF_5**	Reggio Emilia 2	faecal	Red cow	Hay	Unbound
**RE2_CF_6**	Reggio Emilia 2	faecal	Red cow	Hay	Unbound
**RE2_CF_7**	Reggio Emilia 2	faecal	Red cow	Hay	Unbound
**RE2_CF_8**	Reggio Emilia 2	faecal	Red cow	Hay	Unbound
**RE2_CF_9**	Reggio Emilia 2	faecal	Red cow	Hay	Unbound
**RE2_CF_10**	Reggio Emilia 2	faecal	Red cow	Hay	Unbound
**RE2_MIL_1**	Reggio Emilia 2	Milk	Red cow	Hay	Unbound
**RE2_MIL_2**	Reggio Emilia 2	Milk	Red cow	Hay	Unbound
**RE2_MIL_3**	Reggio Emilia 2	Milk	Red cow	Hay	Unbound
**RE2_MIL_4**	Reggio Emilia 2	Milk	Red cow	Hay	Unbound
**RE2_MIL_5**	Reggio Emilia 2	Milk	Red cow	Hay	Unbound
**RE2_MIL_6**	Reggio Emilia 2	Milk	Red cow	Hay	Unbound
**RE2_MIL_7**	Reggio Emilia 2	Milk	Red cow	Hay	Unbound
**RE2_MIL_8**	Reggio Emilia 2	Milk	Red cow	Hay	Unbound
**RE2_MIL_9**	Reggio Emilia 2	Milk	Red cow	Hay	Unbound
**RE2_MIL_10**	Reggio Emilia 2	Milk	Red cow	Hay	Unbound
**RE2_LIT_1**	Reggio Emilia 2	Litter			
**RE2_LIT_2**	Reggio Emilia 2	Litter			
**RE2_LIT_3**	Reggio Emilia 2	Litter			
**RE2_LIT_4**	Reggio Emilia 2	Litter			
**RE2_LIT_5**	Reggio Emilia 2	Litter			
**RE2_LIT_6**	Reggio Emilia 2	Litter			
**RE2_LIT_7**	Reggio Emilia 2	Litter			
**RE2_LIT_8**	Reggio Emilia 2	Litter			
**RE2_LIT_9**	Reggio Emilia 2	Litter			
**RE2_LIT_10**	Reggio Emilia 2	Litter			
**RE2_PC_1**	Reggio Emilia 2	Parmesan Cheese	Red cow	Hay	Unbound
**RE2_PC_2**	Reggio Emilia 2	Parmesan Cheese	Red cow	Hay	Unbound
**RE2_PC_3**	Reggio Emilia 2	Parmesan Cheese	Red cow	Hay	Unbound

Supplementary Table 2: Quality-filtering table of 16S rRNA gene profiling datasets.

Sample	Production site	Matrix	Number of sequenced reads	Number of reads with mean quality > 20	Denoised reads	Chimeric reads	Final Read Number
**P1_CF_1**	Parma 1	faecal	78774	72829	72829	2830	69999
**P1_CF_2**	Parma 1	faecal	77401	73155	73155	2156	70999
**P1_CF_3**	Parma 1	faecal	77380	71315	71315	2290	69025
**P1_CF_4**	Parma 1	faecal	81958	76644	76644	3868	72776
**P1_CF_5**	Parma 1	faecal	96902	88205	88205	3331	84874
**P1_CF_6**	Parma 1	faecal	84537	78688	78688	2770	75918
**P1_CF_7**	Parma 1	faecal	80798	73951	73951	2604	71347
**P1_CF_8**	Parma 1	faecal	82687	78621	78621	4278	74343
**P1_CF_9**	Parma 1	faecal	84688	77222	77222	2659	74563
**P1_CF_10**	Parma 1	faecal	88338	83495	83495	2888	80607
**P1_MIL_1**	Parma 1	Milk	79253	79310	79310	3059	76251
**P1_MIL_2**	Parma 1	Milk	63253	75104	75104	4817	70287
**P1_MIL_3**	Parma 1	Milk	89009	77711	77711	6645	71066
**P1_MIL_4**	Parma 1	Milk	89527	68858	68858	1283	67575
**P1_MIL_5**	Parma 1	Milk	103646	94589	94589	9275	85314
**P1_MIL_6**	Parma 1	Milk	95993	78349	78349	915	77434
**P1_MIL_7**	Parma 1	Milk	105813	94612	94612	5884	88728
**P1_MIL_8**	Parma 1	Milk	92113	85267	85267	3795	81472
**P1_MIL_9**	Parma 1	Milk	94718	84605	84605	5253	79352
**P1_MIL_10**	Parma 1	Milk	94701	86113	86113	7251	78862
**P1_LIT_1**	Parma 1	Litter	72603	66073	66073	2802	63271
**P1_LIT_2**	Parma 1	Litter	92108	84233	84233	3795	80438
**P1_LIT_3**	Parma 1	Litter	103800	92607	92607	3360	89247
**P1_LIT_4**	Parma 1	Litter	81767	75032	75032	6748	68284
**P1_LIT_5**	Parma 1	Litter	109203	97833	97833	2512	95321
**P1_LIT_6**	Parma 1	Litter	114476	103180	103180	4031	99149
**P1_LIT_7**	Parma 1	Litter	102756	92364	92364	2560	89804
**P1_LIT_8**	Parma 1	Litter	84344	78783	78783	6111	72672
**P1_LIT_9**	Parma 1	Litter	106900	96906	96906	3333	93573
**P1_LIT_10**	Parma 1	Litter	102813	91878	91878	3946	87932
**P1_PC_1**	Parma 1	Parmesan Cheese	67927	62348	62348	10167	52181
**P1_PC_2**	Parma 1	Parmesan Cheese	85344	79271	79271	8497	70774
**P1_PC_3**	Parma 1	Parmesan Cheese	68710	62840	62840	8598	54242
**P2_CF_1**	Parma 2	faecal	87450	81175	81175	4340	76835
**P2_CF_2**	Parma 2	faecal	76813	72475	72475	3638	68837
**P2_CF_3**	Parma 2	faecal	75101	71623	71623	2816	68807
**P2_CF_4**	Parma 2	faecal	70484	67140	67140	2619	64521
**P2_CF_5**	Parma 2	faecal	77298	71499	71499	2827	68672
**P2_CF_6**	Parma 2	faecal	82026	76551	76551	4318	72233
**P2_CF_7**	Parma 2	faecal	79731	70424	70424	3257	67167
**P2_CF_8**	Parma 2	faecal	76632	72630	72630	4494	68136
**P2_CF_9**	Parma 2	faecal	76644	72321	72321	3409	68912

**P2_CF_10**	Parma 2	faecal	86479	82055	82055	5107	76948
**P2_MIL_1**	Parma 2	Milk	104711	70116	70116	1091	69025
**P2_MIL_2**	Parma 2	Milk	86450	69386	69386	2035	67351
**P2_MIL_3**	Parma 2	Milk	78719	66238	66238	609	65629
**P2_MIL_4**	Parma 2	Milk	86986	74984	74984	1067	73917
**P2_MIL_5**	Parma 2	Milk	86812	61801	61801	170	61631
**P2_MIL_6**	Parma 2	Milk	89686	64166	64166	1517	62649
**P2_MIL_7**	Parma 2	Milk	106779	88043	88043	1168	86875
**P2_MIL_8**	Parma 2	Milk	79107	69529	69529	3917	65612
**P2_MIL_9**	Parma 2	Milk	79386	70097	70097	7302	62795
**P2_MIL_10**	Parma 2	Milk	78766	69706	69706	6628	63078
**P2_LIT_1**	Parma 2	Litter	73387	68391	68391	2930	65461
**P2_LIT_2**	Parma 2	Litter	65911	62467	62467	1481	60986
**P2_LIT_3**	Parma 2	Litter	102916	98041	98041	3620	94421
**P2_LIT_4**	Parma 2	Litter	79949	76350	76350	4102	72248
**P2_LIT_5**	Parma 2	Litter	71313	68152	68152	1599	66553
**P2_LIT_6**	Parma 2	Litter	63274	59145	59145	1657	57488
**P2_LIT_7**	Parma 2	Litter	83238	78418	78418	4285	74133
**P2_LIT_8**	Parma 2	Litter	89330	83324	83324	884	82440
**P2_LIT_9**	Parma 2	Litter	76203	71673	71673	2257	69416
**P2_LIT_10**	Parma 2	Litter	140909	134090	134090	11866	122224
**P2_PC_1**	Parma 2	Parmesan Cheese	87214	82377	82377	10613	71764
**P2_PC_2**	Parma 2	Parmesan Cheese	70579	67297	67297	9058	58239
**P2_PC_3**	Parma 2	Parmesan Cheese	71218	67459	67459	9447	58012
**P3_CF_1**	Parma 3	faecal	59946	46148	46148	3253	42895
**P3_CF_2**	Parma 3	faecal	53921	37503	37503	3242	34261
**P3_CF_3**	Parma 3	faecal	90545	38875	38875	2973	35902
**P3_CF_4**	Parma 3	faecal	64028	51721	51721	5095	46626
**P3_CF_5**	Parma 3	faecal	67288	47563	47563	5040	42523
**P3_CF_6**	Parma 3	faecal	54215	37028	37028	1515	35513
**P3_CF_7**	Parma 3	faecal	48765	36431	36431	2777	33654
**P3_CF_8**	Parma 3	faecal	55564	36242	36242	3344	32898
**P3_CF_9**	Parma 3	faecal	64996	26131	26131	2511	23620
**P3_CF_10**	Parma 3	faecal	53689	43507	43507	5984	37523
**P3_MIL_1**	Parma 3	Milk	43625	35653	35653	2405	33248
**P3_MIL_2**	Parma 3	Milk	61933	27679	27679	1703	25976
**P3_MIL_3**	Parma 3	Milk	46912	44603	44603	657	43946
**P3_MIL_4**	Parma 3	Milk	64014	26201	26201	505	25696
**P3_MIL_5**	Parma 3	Milk	10993	1173	1173	22	1151
**P3_MIL_6**	Parma 3	Milk	54540	53226	53226	1642	51584
**P3_MIL_7**	Parma 3	Milk	16502	12236	12236	260	11976
**P3_MIL_8**	Parma 3	Milk	70591	9734	9734	136	9598
**P3_MIL_9**	Parma 3	Milk	72158	30751	30751	785	29966
**P3_MIL_10**	Parma 3	Milk	64849	29243	29243	2173	27070
**P3_LIT_1**	Parma 3	Litter	55111	52187	52187	7271	44916

**P3_LIT_2**	Parma 3	Litter	51816	48209	48209	3988	44221
**P3_LIT_3**	Parma 3	Litter	58080	54126	54126	16772	37354
**P3_LIT_4**	Parma 3	Litter	44520	42019	42019	5240	36779
**P3_LIT_5**	Parma 3	Litter	53455	50373	50373	14411	35962
**P3_LIT_6**	Parma 3	Litter	57471	53748	53748	8552	45196
**P3_LIT_7**	Parma 3	Litter	75964	71587	71587	12291	59296
**P3_LIT_8**	Parma 3	Litter	61009	57597	57597	4938	52659
**P3_LIT_9**	Parma 3	Litter	52378	48688	48688	3695	44993
**P3_LIT_10**	Parma 3	Litter	57326	53401	53401	5185	48216
**P3_PC_1**	Parma 3	Parmesan Cheese	41523	32600	32600	4676	27924
**P3_PC_2**	Parma 3	Parmesan Cheese	58337	22358	22358	2385	19973
**P3_PC_3**	Parma 3	Parmesan Cheese	60747	33849	33849	5080	28769
**RE1_CF_1**	Reggio Emilia 1	faecal	71327	50941	50941	4189	46752
**RE1_CF_2**	Reggio Emilia 1	faecal	62554	52380	52380	4179	48201
**RE1_CF_3**	Reggio Emilia 1	faecal	62468	54705	54705	5008	49697
**RE1_CF_4**	Reggio Emilia 1	faecal	57532	54902	54902	5781	49121
**RE1_CF_5**	Reggio Emilia 1	faecal	51793	44971	44971	3613	41358
**RE1_CF_6**	Reggio Emilia 1	faecal	68040	50481	50481	4025	46456
**RE1_CF_7**	Reggio Emilia 1	faecal	61049	54467	54467	3728	50739
**RE1_CF_8**	Reggio Emilia 1	faecal	69710	42128	42128	1997	40131
**RE1_CF_9**	Reggio Emilia 1	faecal	54805	48442	48442	4530	43912
**RE1_CF_10**	Reggio Emilia 1	faecal	77102	68699	68699	6452	62247
**RE1_MIL_1**	Reggio Emilia 1	Milk	52384	17875	17875	466	17409
**RE1_MIL_2**	Reggio Emilia 1	Milk	77090	28236	28236	604	27632
**RE1_MIL_3**	Reggio Emilia 1	Milk	70540	45068	45068	1093	43975
**RE1_MIL_4**	Reggio Emilia 1	Milk	48963	34726	34726	1035	33691
**RE1_MIL_5**	Reggio Emilia 1	Milk	66884	36890	36890	937	35953
**RE1_MIL_6**	Reggio Emilia 1	Milk	61615	16961	16961	279	16682
**RE1_MIL_7**	Reggio Emilia 1	Milk	49298	42948	42948	501	42447
**RE1_MIL_8**	Reggio Emilia 1	Milk	56383	45681	45681	638	45043
**RE1_MIL_9**	Reggio Emilia 1	Milk	57338	25301	25301	627	24674
**RE1_MIL_10**	Reggio Emilia 1	Milk	68195	32900	32900	638	32262
**RE1_LIT_1**	Reggio Emilia 1	Litter	56593	53565	53565	10784	42781
**RE1_LIT_2**	Reggio Emilia 1	Litter	59650	56215	56215	10633	45582
**RE1_LIT_3**	Reggio Emilia 1	Litter	57331	55293	55293	7476	47817
**RE1_LIT_4**	Reggio Emilia 1	Litter	46438	44019	44019	8323	35696
**RE1_LIT_5**	Reggio Emilia 1	Litter	48458	46335	46335	10346	35989
**RE1_LIT_6**	Reggio Emilia 1	Litter	52818	50749	50749	7571	43178
**RE1_LIT_7**	Reggio Emilia 1	Litter	55835	52704	52704	11479	41225
**RE1_LIT_8**	Reggio Emilia 1	Litter	34313	32822	32822	5437	27385
**RE1_LIT_9**	Reggio Emilia 1	Litter	57583	55522	55522	3681	51841
**RE1_LIT_10**	Reggio Emilia 1	Litter	55760	53665	53665	7847	45818
**RE1_PC_1**	Reggio Emilia 1	Parmesan Cheese	47596	40702	40702	9956	30746
**RE1_PC_2**	Reggio Emilia 1	Parmesan Cheese	85695	51179	51179	5816	45363
**RE1_PC_3**	Reggio Emilia 1	Parmesan Cheese	58137	50793	50793	12936	37857

**RE2_CF_1**	Reggio Emilia 2	faecal	77032	67899	67899	2210	65689
**RE2_CF_2**	Reggio Emilia 2	faecal	78315	70992	70992	2430	68562
**RE2_CF_3**	Reggio Emilia 2	faecal	66571	48325	48325	1469	46856
**RE2_CF_4**	Reggio Emilia 2	faecal	74439	55281	55281	2497	52784
**RE2_CF_5**	Reggio Emilia 2	faecal	73675	60608	60608	1913	58695
**RE2_CF_6**	Reggio Emilia 2	faecal	69338	61927	61927	3034	58893
**RE2_CF_7**	Reggio Emilia 2	faecal	80506	54785	54785	2124	52661
**RE2_CF_8**	Reggio Emilia 2	faecal	63840	31015	31015	1344	29671
**RE2_CF_9**	Reggio Emilia 2	faecal	60168	57327	57327	2449	54878
**RE2_CF_10**	Reggio Emilia 2	faecal	53251	49168	49168	3827	45341
**RE2_MIL_1**	Reggio Emilia 2	Milk	54464	45391	45391	518	44873
**RE2_MIL_2**	Reggio Emilia 2	Milk	57466	52807	52807	4420	48387
**RE2_MIL_3**	Reggio Emilia 2	Milk	48404	44322	44322	4206	40116
**RE2_MIL_4**	Reggio Emilia 2	Milk	69597	61771	61771	2346	59425
**RE2_MIL_5**	Reggio Emilia 2	Milk	46915	41655	41655	1350	40305
**RE2_MIL_6**	Reggio Emilia 2	Milk	72485	51295	51295	2953	48342
**RE2_MIL_7**	Reggio Emilia 2	Milk	50773	36956	36956	1915	35041
**RE2_MIL_8**	Reggio Emilia 2	Milk	52980	44040	44040	483	43557
**RE2_MIL_9**	Reggio Emilia 2	Milk	53605	47389	47389	1872	45517
**RE2_MIL_10**	Reggio Emilia 2	Milk	55137	45489	45489	382	45107
**RE2_LIT_1**	Reggio Emilia 2	Litter	70941	66936	66936	1424	65512
**RE2_LIT_2**	Reggio Emilia 2	Litter	70680	66245	66245	2594	63651
**RE2_LIT_3**	Reggio Emilia 2	Litter	77145	70298	70298	459	69839
**RE2_LIT_4**	Reggio Emilia 2	Litter	66400	61805	61805	2696	59109
**RE2_LIT_5**	Reggio Emilia 2	Litter	71428	66486	66486	332	66154
**RE2_LIT_6**	Reggio Emilia 2	Litter	75226	68962	68962	1696	67266
**RE2_LIT_7**	Reggio Emilia 2	Litter	68642	64760	64760	303	64457
**RE2_LIT_8**	Reggio Emilia 2	Litter	74696	69821	69821	722	69099
**RE2_LIT_9**	Reggio Emilia 2	Litter	71044	67325	67325	1861	65464
**RE2_LIT_10**	Reggio Emilia 2	Litter	77393	72620	72620	3922	68698
**RE2_PC_1**	Reggio Emilia 2	Parmesan Cheese	47693	41261	41261	3934	37327
**RE2_PC_2**	Reggio Emilia 2	Parmesan Cheese	48346	31533	31533	5067	26466
**RE2_PC_3**	Reggio Emilia 2	Parmesan Cheese	60244	47658	47658	4826	42832

Supplementary Table 3: Quality-filtering table of bifidobacterial ITS profiling datasets.

Sample	Production site	Matrix	Number of sequenced pe reads	Number of pe reads with mean quality > 20	Number of merged pe reads	No bacterial sequences	Ambiguous bases	Homopolymers > 7	Mismatch in primers >1	Reverse primer not found	Final Read Number
**P1_CF_1**	Parma 1	faecal	112413	111506	93790	3	0	1	1393	715	91678
**P1_CF_2**	Parma 1	faecal	113125	112135	96164	0	0	20	1370	771	94003
**P1_CF_3**	Parma 1	faecal	119972	118745	95180	5	0	5	1453	714	93003
**P1_CF_4**	Parma 1	faecal	119236	114776	100226	1	0	15	1398	878	97934
**P1_CF_5**	Parma 1	faecal	161886	160210	130422	2	0	34	1950	1122	127314
**P1_CF_6**	Parma 1	faecal	128627	127445	107201	0	0	18	1562	675	104946
**P1_CF_7**	Parma 1	faecal	96246	93467	80170	0	0	9	1253	725	78183
**P1_CF_8**	Parma 1	faecal	103155	102438	86540	1	0	2	1194	699	84644
**P1_CF_9**	Parma 1	faecal	75867	53808	46440	0	0	5	814	477	45144
**P1_CF_10**	Parma 1	faecal	131839	130549	108238	0	0	21	1640	696	105881
**P1_MIL_1**	Parma 1	Milk	65572	64263	54196	0	0	13	933	398	52852
**P1_MIL_2**	Parma 1	Milk	48776	47394	39876	0	0	12	592	353	38919
**P1_MIL_3**	Parma 1	Milk	68393	66791	50645	0	0	12	1036	456	49141
**P1_MIL_4**	Parma 1	Milk	73965	73110	57390	0	0	8	966	473	55943
**P1_MIL_5**	Parma 1	Milk	84680	81706	69452	0	0	21	1203	658	67570
**P1_MIL_6**	Parma 1	Milk	72378	71403	56559	42	0	10	912	531	55064
**P1_MIL_7**	Parma 1	Milk	127548	125801	102588	0	0	14	1856	806	99912
**P1_MIL_8**	Parma 1	Milk	104062	102846	81473	0	0	62	1438	584	79389
**P1_MIL_9**	Parma 1	Milk	43835	43060	34796	1	0	6	826	327	33636
**P1_MIL_10**	Parma 1	Milk	99402	98385	78986	9	0	10	1059	572	77336
**P1_PC_1**	Parma 1	Parmesan Cheese	80778	79954	68894	1	1	3	1246	753	66890
**P1_PC_2**	Parma 1	Parmesan Cheese	110832	109165	85963	3	0	2	1730	863	83365
**P1_PC_3**	Parma 1	Parmesan Cheese	123825	122489	97597	0	0	0	1870	980	94747
**P2_CF_1**	Parma 2	faecal	76438	75603	65712	1	0	3	937	590	64181
**P2_CF_2**	Parma 2	faecal	88293	86655	74691	2	0	14	1093	618	72964
**P2_CF_3**	Parma 2	faecal	104069	102570	81358	6	0	48	1316	698	79290
**P2_CF_4**	Parma 2	faecal	78379	76305	65358	3	0	29	884	556	63886
**P2_CF_5**	Parma 2	faecal	115281	111492	98482	2	0	29	1396	819	96236
**P2_CF_6**	Parma 2	faecal	80515	79523	64254	0	0	26	936	510	62782
**P2_CF_7**	Parma 2	faecal	102689	101592	85286	1	0	24	1158	676	83427
**P2_CF_8**	Parma 2	faecal	91087	89213	77840	0	0	5	1295	619	75921
**P2_CF_9**	Parma 2	faecal	104982	103359	82484	6	0	26	1240	708	80504
**P2_CF_10**	Parma 2	faecal	109645	107945	87356	0	0	22	1273	718	85343
**P2_MIL_1**	Parma 2	Milk	123199	122011	96830	3	0	20	1536	754	94517
**P2_MIL_2**	Parma 2	Milk	115284	114254	93748	6	0	3	1510	573	91656
**P2_MIL_3**	Parma 2	Milk	108551	107643	89790	3	0	21	1372	766	87628
**P2_MIL_4**	Parma 2	Milk	103622	102002	75058	12	0	6	1488	746	72806

**P2_MIL_5**	Parma 2	Milk	92800	91548	70967	1	0	10	1149	530	69277
**P2_MIL_6**	Parma 2	Milk	127082	125960	103872	0	0	34	1620	877	101341
**P2_MIL_7**	Parma 2	Milk	94015	92677	70892	6	0	15	1391	640	68840
**P2_MIL_8**	Parma 2	Milk	134624	132796	118391	1	0	19	2070	1014	115287
**P2_MIL_9**	Parma 2	Milk	73776	71640	61275	3	0	8	814	462	59988
**P2_MIL_10**	Parma 2	Milk	121755	120341	94670	3	0	4	1529	708	92426
**P2_PC_1**	Parma 2	Parmesan Cheese	75671	74921	61991	8	0	1	884	459	60639
**P2_PC_2**	Parma 2	Parmesan Cheese	79119	78300	65576	7	0	5	902	472	64190
**P2_PC_3**	Parma 2	Parmesan Cheese	122328	120757	94346	4	0	7	1559	630	92146
**P3_CF_1**	Parma 3	faecal	59946	58227	55028	8	0	3	46437	84	8496
**P3_CF_2**	Parma 3	faecal	53921	51817	48719	0	0	1	37909	150	10659
**P3_CF_3**	Parma 3	faecal	90545	83361	75655	45	0	10	39651	340	35609
**P3_CF_4**	Parma 3	faecal	64028	62101	59345	50	0	2	52176	78	7039
**P3_CF_5**	Parma 3	faecal	67288	65535	61692	0	1	11	48051	168	13461
**P3_CF_6**	Parma 3	faecal	54215	51466	47512	138	0	4	36964	92	10314
**P3_CF_7**	Parma 3	faecal	48765	47217	44548	1	0	1	36822	64	7660
**P3_CF_8**	Parma 3	faecal	55564	53535	49933	2	0	5	36704	132	13090
**P3_CF_9**	Parma 3	faecal	64996	60756	54045	1	0	7	26577	293	27167
**P3_CF_10**	Parma 3	faecal	53689	51218	48801	0	0	9	43562	72	5158
**P3_MIL_1**	Parma 3	Milk	43625	42492	39821	2	0	6	35892	61	3860
**P3_MIL_2**	Parma 3	Milk	61933	57848	52574	4	0	2	28227	272	24069
**P3_MIL_3**	Parma 3	Milk	61645	60782	53908	4	1	8	8159	384	45352
**P3_MIL_4**	Parma 3	Milk	64014	63286	58329	0	0	8	26613	232	31476
**P3_MIL_5**	Parma 3	Milk	64573	63980	55747	8	0	4	722	349	54664
**P3_MIL_6**	Parma 3	Milk	57172	48851	38124	14842	0	21	11178	131	11951
**P3_MIL_7**	Parma 3	Milk	82585	66636	54572	15889	1	35	7608	358	30681
**P3_MIL_8**	Parma 3	Milk	70591	66760	56779	1	0	15	10587	415	45761
**P3_MIL_9**	Parma 3	Milk	72158	50798	46650	977	1	2	29949	186	15535
**P3_MIL_10**	Parma 3	Milk	64849	60653	53796	2010	0	8	27110	216	24452
**P3_PC_1**	Parma 3	Parmesan Cheese	41523	40573	38351	2	0	0	33075	39	5235
**P3_PC_2**	Parma 3	Parmesan Cheese	58337	57540	28063	20	0	1	22825	44	5173
**P3_PC_3**	Parma 3	Parmesan Cheese	60747	60273	55239	2	1	0	34447	149	20640
**RE1_CF_1**	Reggio Emilia 1	faecal	71327	69551	66088	2	1	6	51487	129	14463
**RE1_CF_2**	Reggio Emilia 1	faecal	62554	59742	57343	24	1	3	52692	51	4572
**RE1_CF_3**	Reggio Emilia 1	faecal	62468	60740	58362	22	0	3	54989	28	3320
**RE1_CF_4**	Reggio Emilia 1	faecal	77261	52749	45495	0	0	114	813	447	44121
**RE1_CF_5**	Reggio Emilia 1	faecal	51793	50454	48326	48	0	5	45197	29	3047
**RE1_CF_6**	Reggio Emilia 1	faecal	68040	61625	59109	17	0	5	50896	80	8111
**RE1_CF_7**	Reggio Emilia 1	faecal	61049	59670	57541	52	0	6	54697	33	2753
**RE1_CF_8**	Reggio Emilia 1	faecal	69710	60861	57302	25	1	6	42459	166	14645
**RE1_CF_9**	Reggio Emilia 1	faecal	54805	53648	51519	67	0	5	48557	40	2850
**RE1_CF_10**	Reggio Emilia 1	faecal	77102	73673	71726	23	0	38	69095	23	2547
**RE1_MIL_1**	Reggio Emilia 1	Milk	52384	48569	40127	4608	1	10	10888	152	24468
**RE1_MIL_2**	Reggio Emilia 1	Milk	77090	71799	61866	1891	0	3	26875	299	32798
**RE1_MIL_3**	Reggio Emilia 1	Milk	70540	65960	59401	2632	0	10	42081	177	14501

**RE1_MIL_4**	Reggio Emilia 1	Milk	48963	47279	42917	2712	1	9	31127	80	8988
**RE1_MIL_5**	Reggio Emilia 1	Milk	66884	61173	51151	13632	0	12	21791	194	15522
**RE1_MIL_6**	Reggio Emilia 1	Milk	61615	51524	39898	2893	4	16	13079	356	23550
**RE1_MIL_7**	Reggio Emilia 1	Milk	49298	47606	44577	11735	1	6	30521	16	2298
**RE1_MIL_8**	Reggio Emilia 1	Milk	56383	52943	49265	13688	3	5	31300	60	4209
**RE1_MIL_9**	Reggio Emilia 1	Milk	57338	53336	42714	10819	1	34	13303	220	18337
**RE1_MIL_10**	Reggio Emilia 1	Milk	68195	60123	55079	3108	0	7	29756	202	22006
**RE1_PC_1**	Reggio Emilia 1	Parmesan Cheese	47596	46227	44211	33	5	0	41025	29	3119
**RE1_PC_2**	Reggio Emilia 1	Parmesan Cheese	85695	84502	58857	25	0	1	51821	99	6911
**RE1_PC_3**	Reggio Emilia 1	Parmesan Cheese	58137	56870	54676	77	1	0	51206	34	3358
**RE2_CF_1**	Reggio Emilia 2	faecal	77032	76593	72606	16	0	5	66554	51	5980
**RE2_CF_2**	Reggio Emilia 2	faecal	78970	50994	42989	0	0	2	868	412	41707
**RE2_CF_3**	Reggio Emilia 2	faecal	66571	66085	61498	6	0	4	47545	134	13809
**RE2_CF_4**	Reggio Emilia 2	faecal	74439	66762	61528	357	0	4	53006	70	8091
**RE2_CF_5**	Reggio Emilia 2	faecal	73675	69281	65044	226	0	5	58905	51	5857
**RE2_CF_6**	Reggio Emilia 2	faecal	69338	68270	64412	16	0	8	60678	41	3669
**RE2_CF_7**	Reggio Emilia 2	faecal	80506	77153	69467	335	0	7	54587	162	14376
**RE2_CF_8**	Reggio Emilia 2	faecal	63840	55602	48302	136	0	6	31180	181	16799
**RE2_CF_9**	Reggio Emilia 2	faecal	67247	61377	55793	3808	0	10	43940	81	7956
**RE2_CF_10**	Reggio Emilia 2	faecal	88315	65235	53580	0	0	3	886	516	52175
**RE2_MIL_1**	Reggio Emilia 2	Milk	54464	53703	50933	1	0	4	45803	55	5070
**RE2_MIL_2**	Reggio Emilia 2	Milk	57466	57006	54981	0	1	0	53373	25	1582
**RE2_MIL_3**	Reggio Emilia 2	Milk	48404	47954	46228	0	0	0	44732	10	1486
**RE2_MIL_4**	Reggio Emilia 2	Milk	69597	68558	65763	1	1	18	62329	33	3381
**RE2_MIL_5**	Reggio Emilia 2	Milk	46915	46370	44483	1	0	2	41994	25	2461
**RE2_MIL_6**	Reggio Emilia 2	Milk	72485	63663	61457	0	0	12	51645	90	9710
**RE2_MIL_7**	Reggio Emilia 2	Milk	50773	50469	46688	0	1	0	37109	66	9512
**RE2_MIL_8**	Reggio Emilia 2	Milk	52980	52461	49689	6	1	4	44638	61	4979
**RE2_MIL_9**	Reggio Emilia 2	Milk	53605	53068	50808	0	0	5	47983	28	2792
**RE2_MIL_10**	Reggio Emilia 2	Milk	55137	54184	51655	1	0	23	45871	58	5702
**RE2_PC_1**	Reggio Emilia 2	Parmesan Cheese	47693	47232	45400	8	4	0	41731	33	3624
**RE2_PC_2**	Reggio Emilia 2	Parmesan Cheese	48346	47893	33649	35	0	0	31699	22	1893
**RE2_PC_3**	Reggio Emilia 2	Parmesan Cheese	60244	59264	55566	4	1	0	48164	74	7323

Supplementary Table 4: Cow DNA filtering of shotgun metagenomics datasets.

Sample	Sequenced reads	Cow-filtered reads
P1_PC_1	37612529	37595526
P2_PC_1	25088478	21816623
P1_LIT_4	13703634	13682174
P2_LIT_8	12273062	12244806
P2_MIL_4	14200899	4976043
P2_MIL_8	19144692	7033929
P3_CF_9	14590125	14576473
RE1_CF_1	17439821	17437711

Supplementary Table 5: Primers used to test strains transmission across the Parmesan cheese production chain

Species	Forward Primer	Reverse Primer
Pseudoflavonifractor capillosus	CGATGCAGAAGGAACCAAGG	GGGTTGCATTTTCAATCGCG
Treponema porcinum	TCTGGACTTTTGAACATGGAGC	CAGCACGCTTGAAACTGGTA
Prevotella ruminicola	GATTTCCCGTTCCGTGCTTG	TCGAACCGTAAACTCTGCCT
Paraprevotella clara	TCCATCAGCACTCCCATTGT	ATGCGTAACGTAGGGTGTCA
Corynebacterium stationis	CTCAATATCAGCCGCGGAAC	TTTCTGTATCACCGGCAAGC
Oligella ureolytica	ACGCACACTGAGACTGCAAG	TGGTGGTACTGCTGGAGTTG
Jeotgallicoccus psychrophilus	TGTCTGATGACGACCGCTAC	CCGGTGACACTGTTAATTGC
Atopostipes suicloacalis	AACTTGGTCACTCCGTCATTG	TCGCCACTTCAACATTGTGT
Pseudoclavibacter soli	TCTGAACAGACTGCGACTGC	AATCTGCTCTGCGATGTCCT
Kocuria kristinae	ACGAGGTCTACGAGGTGCTG	TCTGGTCTATGTCGCTGGTC
Corynebacterium variable	GAGGAATCTTCCTGCGAATG	GCATTGTGCGAGTAGCTGAA
Lactobacillus delbrueckii (P2)	AAGCAAGACCATAAGCAAGC	TGCCGTTACATCAAGGTTCC
Lactobacillus delbrueckii (P1)	GAACACTCTGGCTCTGCAAC	TTGGTTGCCGATGATTTGCA
Streptococcus thermophilus	GACCACCTTCAACTCCTCCA	ATAGTTGGCAATTCCGTGCT
Lactobacillus helveticus	CAGCGAGGACGTGTGAATAC	TTGTACCAACTTGACGCATATAA
Bifidobacterium mongoliense (species-specific)	CGTTCGATGTGGTGCTTCAT	AACCATCATGCCTCCCCATC
Bifidobacterium mongoliense (strain-specific)	TACCATTCCACCGTCAAACG	AAAGGCGAACTTGGCGAGAC

Supplementary Table 6: Shotgun metagenomics reads mapping using MetaSNV

**Shotgun dataset mapped**	**Cheesemaking site where the genome was assembled**	**Genome**	**Average Genome Coverage**
**P1_PC_1**	P2	*Atopostipes suicloacalis*	0.0000
	**P1**	*Bifidobacterium mongoliense*	1.8661
	**P1**	*Corynebacterium stationis*	0.0022
	**P1**	*Corynebacterium variabile*	2.0206
	P2	*Jeotgalicoccus psychrophilus*	0.0000
	P2	*Lactobacillus delbrueckii*	0.0000
	P2	*Lactobacillus helveticus*	0.0000
	P2	*Oligella ureolytica*	0.0000
	**P1**	*Pseudoclavibacter soli*	6.7860
	P2	*Streptococcus thermophilus*	0.0000
**P2_PC_1**	**P2**	*Atopostipes suicloacalis*	0.0003
	P1	*Bifidobacterium mongoliense*	0.0000
	P1	*Corynebacterium stationis*	0.0000
	P1	*Corynebacterium variabile*	0.0000
	**P2**	*Jeotgalicoccus psychrophilus*	0.0009
	**P2**	*Lactobacillus delbrueckii*	100.7681
	**P2**	*Lactobacillus helveticus*	451.2940
	**P2**	*Oligella ureolytica*	0.0004
	P1	*Pseudoclavibacter soli*	0.0000
	**P2**	*Streptococcus thermophilus*	80.0155

Supplementary Table 7: *B. mongoliense* BMONG18 genes overespressed (> 3 fold) during growth on milk respect to MRS + glucose.

ORFs	MRS + Glucose (Average RPKM)	MILK (Average RPKM)	stDEV MRS + Glucose	stDEV MILK	Fold Increase in MILK respect to [illegible]	ANOVA p-value	Functional Annotation
BMONG18_0277	5.22	148.33	5.57834396	40.49570133	27.4	0.004	pilus assembly protein TadE
BMONG18_0278	10.78	148.45	3.635073452	29.59783915	12.8	0.001	pilus biosynthesis protein TadE
BMONG18_0279	14.86	74.01	4.935472554	13.62964959	4.0	0.002	hypothetical protein
BMONG18_0280	5.00	211.43	3.764094048	48.76072418	41.3	0.002	type II secretion system protein, pilus assembly
BMONG18_0281	9.23	378.26	5.042041947	72.08882599	40.0	0.001	Flp pilus assembly protein
BMONG18_0282	9.11	281.41	1.411644077	58.05949244	29.9	0.001	type IV secretion system protein
BMONG18_0069	5.62	114.35	1.78267103	20.62538303	19.4	0.001	glucodextranase
BMONG18_0138	6.82	113.44	4.261465632	39.90216313	15.6	0.010	glycosil transferase
BMONG18_0464	23.72	159.68	3.34246082	28.58593141	5.7	0.001	alpha-acetolactate decarboxylase
BMONG18_0630	17.08	76.35	6.828910772	11.43190016	3.5	0.002	beta-galactosidase
BMONG18_0670	8.41	152.67	4.404553818	54.32421017	17.2	0.010	xylose isomerase
BMONG18_0671	8.51	141.20	0.792785175	29.17351806	15.6	0.001	xylose isomerase
BMONG18_1027	6.61	123.72	2.077225072	22.48313782	17.7	0.001	glycosyl transferase
BMONG18_1133	26.65	177.33	5.244837684	31.92936646	5.7	0.001	aryl-phospho-beta-D-glucosidase
BMONG18_1137	15.93	108.49	1.535193473	14.6699301	5.8	0.000	glycoside hydrolase
BMONG18_1144	12.97	90.37	3.128739416	21.87464576	6.0	0.004	alpha-galactosidase
BMONG18_1274	15.13	85.66	2.555441449	15.19882295	4.7	0.001	beta-fructofuranosidase
BMONG18_1385	5.78	58.62	0.961671981	12.28308253	9.1	0.002	beta-mannosidase
BMONG18_1393	32.49	126.04	1.200825133	11.95696907	2.9	0.000	exo-alpha-sialidase
BMONG18_1481	2.30	35.09	2.37092858	6.705916716	14.2	0.001	glycosyl transferase family 9
BMONG18_1717	20.90	244.98	4.563583059	35.76583356	10.7	0.000	alpha-galactosidase
BMONG18_0006	14.52	392.43	4.49395405	65.88716768	26.0	0.001	major facilitator transporter
BMONG18_0049	56.03	225.94	7.498581888	37.38926761	3.0	0.002	gluconate transporter
BMONG18_0065	55.36	265.00	15.97760235	29.45440905	3.8	0.000	sugar ABC transporter permease
BMONG18_0066	39.57	298.56	12.23812291	44.95495986	6.5	0.001	sugar ABC transporter permease
BMONG18_0068	36.36	161.84	7.827247111	17.6277879	3.5	0.000	ABC transporter substrate-binding protein
BMONG18_0105	25.24	110.76	2.045177335	15.39611519	3.4	0.001	sugar ABC transporter ATPase
BMONG18_0121	32.47	137.72	4.407304883	5.590407349	3.2	0.000	ABC transporter permease
BMONG18_0142	5.04	134.20	1.365861267	28.16078832	25.6	0.001	ABC transporter
BMONG18_0145	16.67	104.00	2.172797045	19.37198215	5.2	0.001	ABC transporter ATP-binding protein
BMONG18_0147	32.73	183.19	0.778821118	24.04870843	4.6	0.000	ABC transporter ATP-binding protein
BMONG18_0150	27.04	258.24	3.468318084	53.30170393	8.5	0.002	ABC transporter permease
BMONG18_0151	13.18	159.06	3.471634245	30.82683056	11.1	0.001	ABC transporter permease
BMONG18_0152	18.00	118.19	5.827671605	21.24581933	5.6	0.001	ABC transporter substrate-binding protein
BMONG18_0182	27.05	193.65	2.353565168	18.81825659	6.2	0.000	MFS transporter
BMONG18_0404	14.73	124.83	3.633616701	14.12531391	7.5	0.000	ABC transporter
BMONG18_0465	42.36	194.93	3.275014707	31.13535825	3.6	0.001	MFS permease
BMONG18_0857	31.95	154.22	1.696082938	18.8982862	3.8	0.000	ABC transporter
BMONG18_0979	6.70	208.40	2.339827558	38.41529802	30.1	0.001	ABC transporter ATP-binding protein

BMONG18_0980	23.75	521.45	4.455087915	76.23802605	21.0	0.000	ABC transporter permease
BMONG18_0981	31.28	297.74	4.714432309	45.52546357	8.5	0.001	ABC transporter permease
BMONG18_1014	73.99	306.66	3.13573362	12.48447966	3.1	0.000	ABC transporter permease
BMONG18_1029	30.94	300.27	8.890936415	50.56465634	8.7	0.001	ABC transporter ATP-binding protein
BMONG18_1061	13.71	190.85	2.315177387	43.02955441	12.9	0.002	permease
BMONG18_1134	30.94	375.41	4.042475149	62.91536275	11.1	0.001	PTS galactitol transporter subunit IIC
BMONG18_1276	17.33	250.01	2.445013701	46.31744557	13.4	0.001	sugar ABC transporter permease
BMONG18_1277	19.02	203.49	6.826106528	32.74822136	9.7	0.001	sugar ABC transporter permease
BMONG18_1305	35.66	386.55	6.531747801	69.78433504	9.8	0.001	ABC transporter ATP-binding protein
BMONG18_1451	46.28	375.44	3.372491858	21.55564035	7.1	0.000	MFS transporter
BMONG18_1498	43.80	309.31	6.760756195	55.68996347	6.1	0.001	ABC transporter ATP-binding protein
BMONG18_1540	61.53	277.48	19.2518127	50.75402095	3.5	0.002	MFS transporter
BMONG18_1733	45.37	237.79	5.674215717	38.11770222	4.2	0.001	ABC 3 transport family
BMONG18_1734	44.68	180.61	3.620746102	28.81166684	3.0	0.001	ABC transporter
BMONG18_1736	15.35	117.28	3.837615709	22.50769443	6.6	0.002	ABC transporter permease
BMONG18_1741	89.69	424.00	6.364522475	50.07007946	3.7	0.000	ABC transporter permease
BMONG18_1743	131.71	798.01	10.35668366	95.12671824	5.1	0.000	ABC transporter ATP-binding protein

Supplementary Table 8: Filtering table of 16S rRNA gene microbial profiling datasets of the 20 individuals enrolled in the study

Sample	Group	input	filtered	denoised	merged	non-chimeric
Human_1_T0	NoMilk	75403	59900	59900	58406	53683
Human_1_T7	NoMilk	46716	19737	19737	18584	18237
Human_1_T14	NoMilk	83857	35052	35052	34135	31969
Human_2_T0	NoMilk	70630	61012	61012	60190	56447
Human_2_T7	NoMilk	86726	39100	39100	38019	34932
Human_2_T14	NoMilk	68319	27838	27838	26546	25539
Human_3_T0	NoMilk	57714	42190	42190	41123	36735
Human_3_T7	NoMilk	83139	37387	37387	36139	34728
Human_3_T14	NoMilk	90693	33386	33386	32129	30366
Human_4_T0	NoMilk	65249	60746	60746	59360	51903
Human_4_T7	NoMilk	62994	26562	26562	25442	23641
Human_4_T14	NoMilk	98187	39375	39375	38134	36147
Human_5_T0	NoMilk	61967	56372	56372	55587	51668
Human_5_T7	NoMilk	74024	23476	23476	22938	21899
Human_5_T14	NoMilk	72695	27941	27941	27486	26249
Human_6_T0	NoMilk	63200	50267	50267	49588	46277
Human_6_T7	NoMilk	63948	26324	26324	25580	22296
Human_6_T14	NoMilk	74330	28037	28037	27329	25531
Human_7_T0	NoMilk	67387	57184	57184	56264	50769
Human_7_T7	NoMilk	71798	28398	28398	27665	24427
Human_7_T14	NoMilk	51931	18409	18409	17843	16100
Human_8_T0	NoMilk	69739	61963	61963	60825	59879
Human_8_T7	NoMilk	118203	51278	51278	48951	46446
Human_8_T14	NoMilk	57681	23717	23717	22846	22542
Human_9_T0	NoMilk	69473	61732	61732	60382	54550
Human_9_T7	NoMilk	181497	71148	71148	68135	61921
Human_9_T14	NoMilk	70628	21170	21170	20244	19238
Human_10_T0	NoMilk	85736	79385	79385	78465	72546
Human_10_T7	NoMilk	76029	68745	68745	65827	61298
Human_10_T14	NoMilk	110574	86093	86093	84624	78365
Human_11_T0	Milk	85729	70931	70931	69878	63444
Human_11_T7	Milk	74927	69265	69265	64837	58212
Human_11_T14	Milk	69274	59275	59275	57937	52735
Human_12_T0	Milk	68374	59986	59986	58051	54059
Human_12_T7	Milk	55189	26156	26156	24752	21982
Human_12_T14	Milk	46386	19697	19697	19067	18361
Human_13_T0	Milk	61957	53337	53337	52570	47402
Human_13_T7	Milk	44752	18734	18734	18031	17071
Human_13_T14	Milk	64328	24928	24928	24348	22683
Human_14_T0	Milk	83200	72623	72623	71128	63300
Human_14_T0	Milk	72547	28206	28206	27029	25119
Human_14_T14	Milk	91100	38649	38649	37608	35467
Human_15_T14	Milk	74676	67920	67920	66616	62529
Human_15_T0	Milk	90147	38652	38652	37555	34950
Human_15_T7	Milk	50179	21983	21983	21253	19999
Human_16_T14	Milk	64733	39688	39688	38421	30388
Human_16_T0	Milk	71072	30195	30195	29035	26797

Human_16_T7	Milk	68755	30238	30238	29434	28157
Human_17_T14	Milk	76141	55594	55594	54508	49288
Human_17_T0	Milk	47306	19961	19961	18935	16235
Human_17_T7	Milk	18148	7875	7875	7250	7003
Human_18_T14	Milk	65117	55916	55916	54328	50860
Human_18_T0	Milk	60020	22682	22682	20783	16357
Human_18_T7	Milk	82064	32527	32527	31488	28679
Human_19_T14	Milk	75146	48939	48939	48143	46481
Human_19_T0	Milk	62704	24980	24980	23579	22819
Human_19_T7	Milk	81723	26432	26432	25653	25151
Human_20_T14	Milk	74916	56040	56040	54821	50769
Human_20_T0	Milk	59717	23454	23454	22902	22274
Human_20_T7	Milk	70346	20371	20371	19800	18984

Supplementary Table 9: Covariances between T7 as well as T10 abundance of *B. mongoliense* BMONG18 and genera observed at T0 in faecal samples of Milk group individuals.

	***Lachnospiraceae* NK4B4 group**	***Subdoligranulum***
**T7**	0.611 (p-value = 0.022)	0..556 (p-value = 0.037)
**T10**	0.722 (p-value = 0.007)	0..667 (p-value = 0.012)

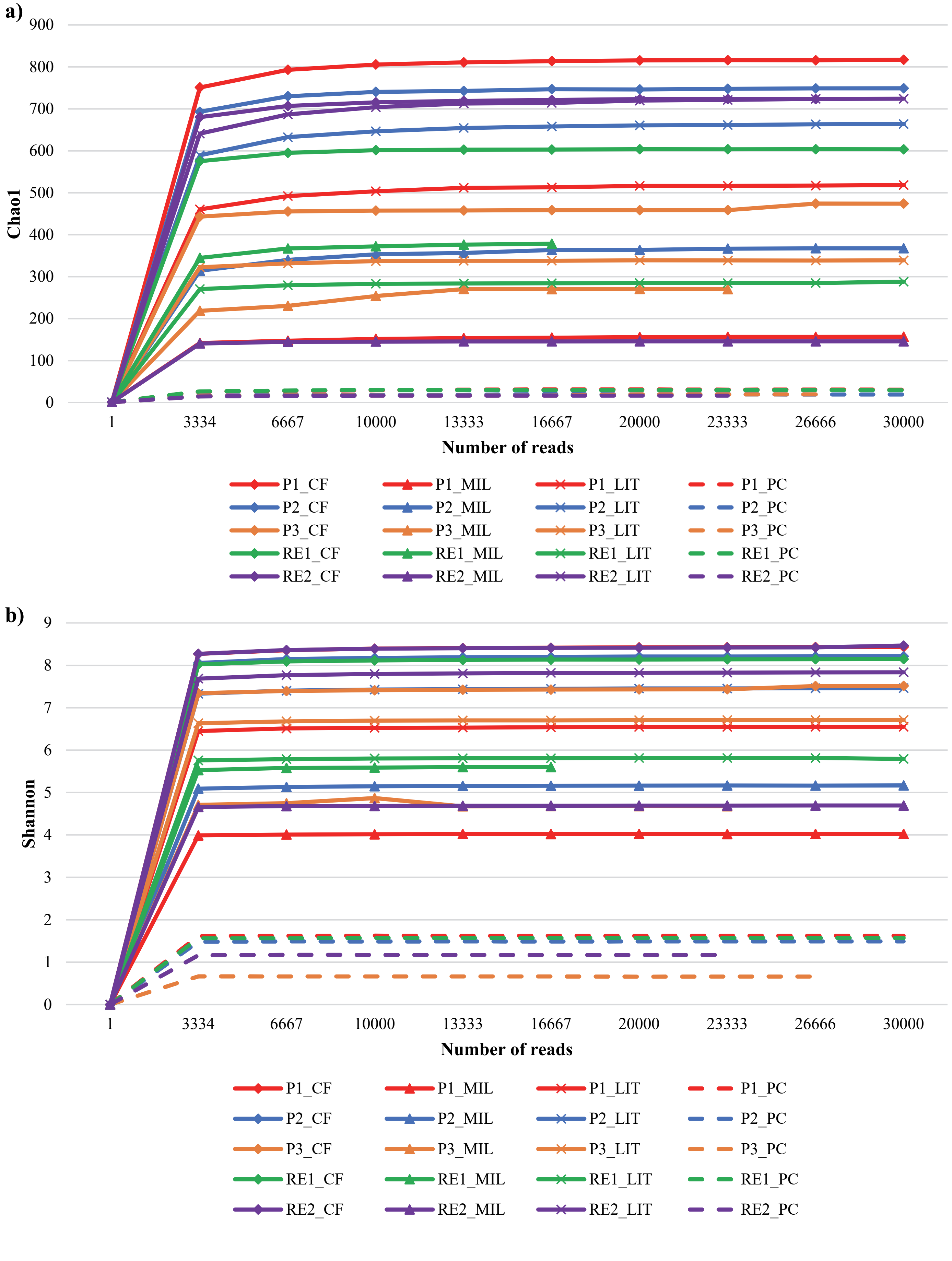

Average alpha-diversity curves of the 165 samples included in this study. The curves reported in the graphs represents the average Chao1 (panel a) and Shannon (panel b) biodiversity indexes observed for each matrix type sampled in the five cheese making sites at 10 subsampling of the whole 16S rRNA gene profiling data normalized at 30,000 reads. Source data are provided as a Source Data file.

Supplementary Figure 1

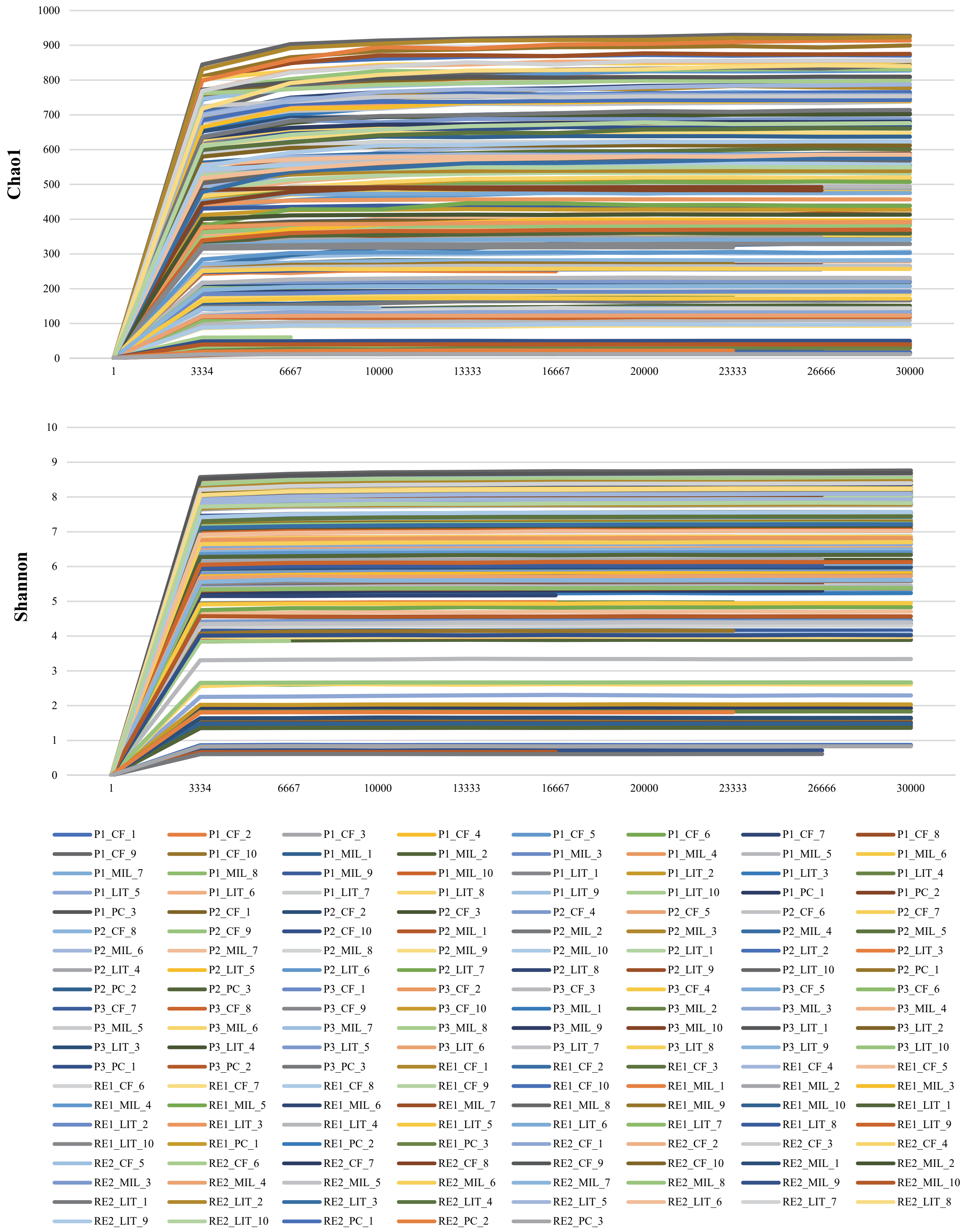

Alpha-diversity curves of all 165 samples included in this study. The curves reported in the graphs represents the Chao1 (panel a) and Shannon (panel b) biodiversity indexes observed at 10 subsampling of the whole 16S rRNA gene profiling data normalized at 30,000 reads. Source data are provided as a Source Data file.

Supplementary Figure 2

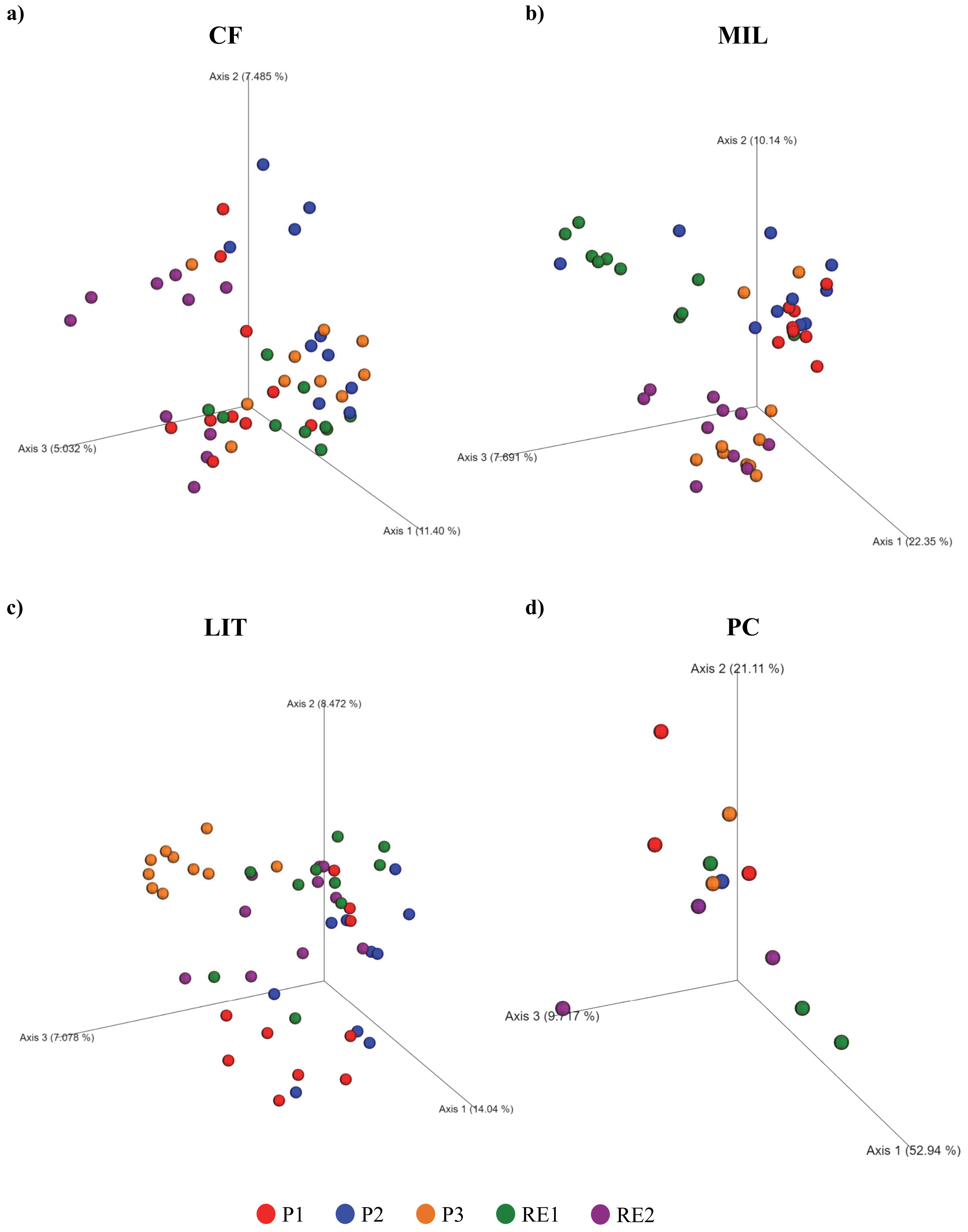

Beta-diversity of all the 165 included in this study grouped by matrix type. Panels a, b, c and d reports a PCoA representation of all the CF, MIL, LIT and PC samples, respectively. Source data are provided as a Source Data file.

Supplementary Figure 3

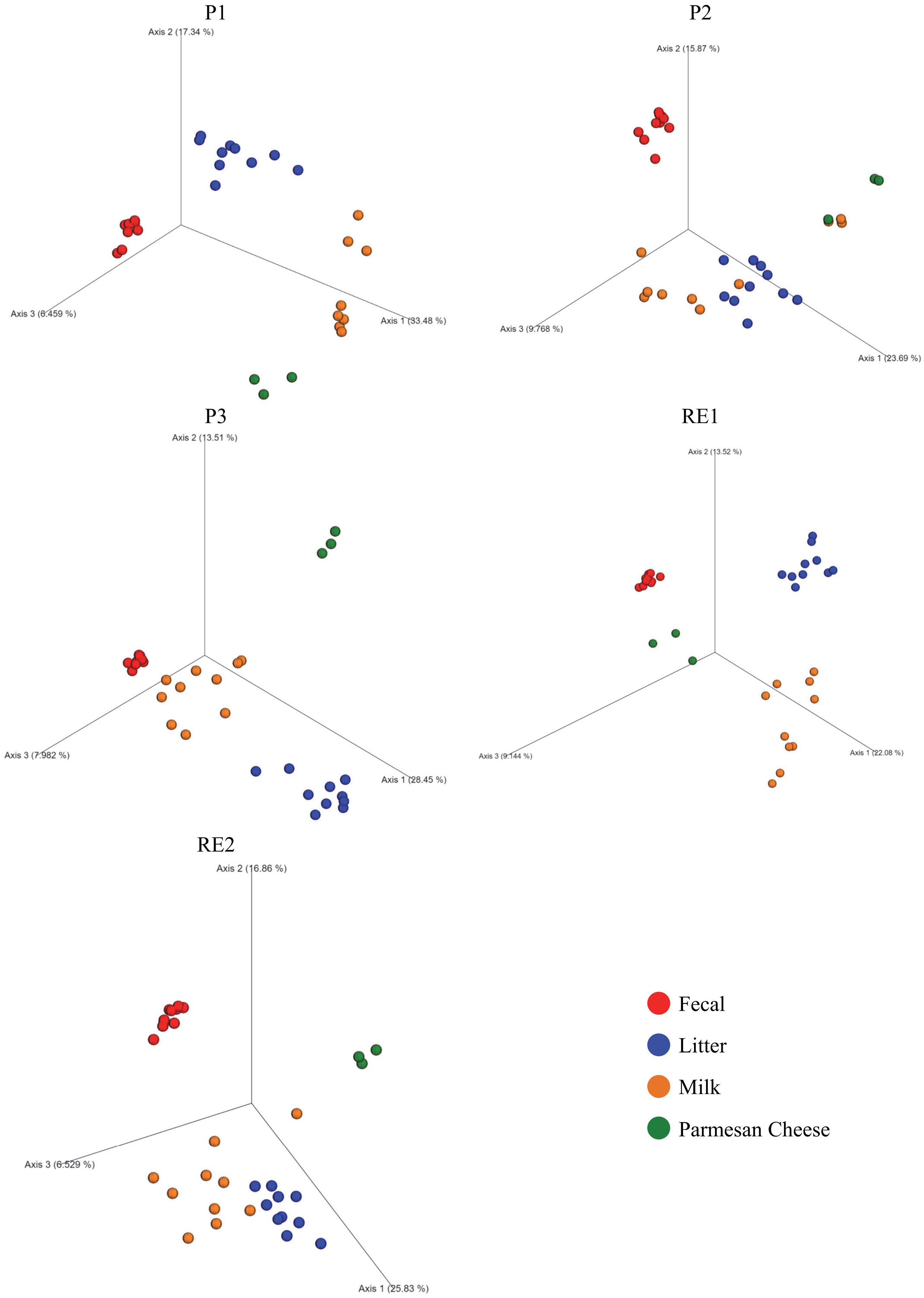

Beta-diversity of all 165 samples included in this study grouped by cheesemaking site. Panels a, b, c, d and e reports a PCoA representation of all the CF, MIL, LIT and PC samples collected from P1, P2, P3, RE1 and RE2 cheesemaking sites, respectively. Source data are provided as a Source Data file.

Supplementary Figure 4

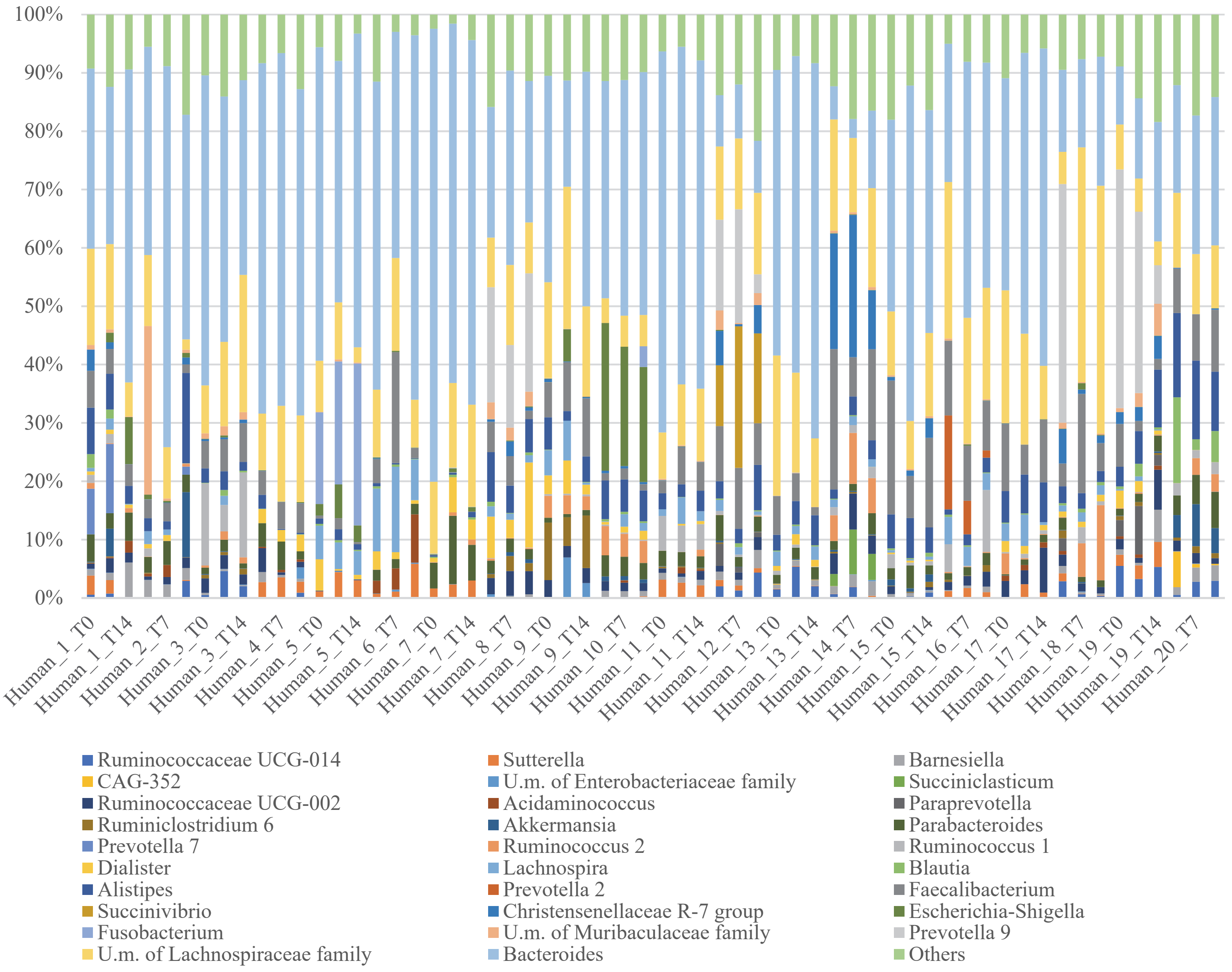

Genus-level taxonomic composition of the gut microbiota composition at T0, T7 and T14 of individuals enrolled in the pilot study. The bar plot shows only genera with relative abundance >5 % in at least a sample. Source data are provided as a Source Data file.

Supplementary Figure 5
