# Supplementary material for: Colonization of the human gut by bovine bacteria present in Parmesan cheese
Source: Nat Commun. 2019 Mar 20;10:1286. doi: 10.1038/s41467-019-09303-w (PMC6426854; doi:10.1038/s41467-019-09303-w)
Supplement: Supplementary file 3 — Description of Additional Supplementary Files [file 41467_2019_9303_MOESM3_ESM.pdf]

Description of Additional Supplementary Files

File Name: Supplementary Data 1
Description: 16S rRNA gene microbial profiling results of cow feces, litters, milk, Parmesan cheese samples. The tables report the profiles obtained at the genus level for each cow feces (CF), litters (LIT), milk (MIL), Parmesan cheese (PC) sample included in this study and the average profiles obtained for each matrix and sampling site.

File Name: Supplementary Data 2
Description: 16S rRNA gene microbial profiling OTUs table of cow feces, litters, milk, Parmesan cheese samples. OTUs table obtained from analysis of all the datasets corresponding to cow feces (CF), litters (LIT), milk (MIL), Parmesan cheese (PC) samples included in this study.

File Name: Supplementary Data 3
Description: Bifidobacterial ITS profiling results of cow feces, litters, milk, Parmesan cheese samples. The tables report the profiles obtained at the species level for each cow feces (CF), litters (LIT), milk (MIL), Parmesan cheese (PC) sample included in this study.

File Name: Supplementary Data 4
Description: SNP profiling results. The tables report the SNP profiles obtained from shotgun metagenomics data of samples collected in cheese making sites P1 and P2.

File Name: Supplementary Data 5
Description: 16S rRNA gene microbial profiling results of the human trial. The tables report the profiles obtained at the genus level and the alpha-diversity of each individual enrolled in the trial study.
