# Supplementary material for: Colonization of the human gut by bovine bacteria present in Parmesan cheese
Source: Nat Commun. 2019 Mar 20;10:1286. doi: 10.1038/s41467-019-09303-w (PMC6426854; doi:10.1038/s41467-019-09303-w)
Supplement: Supplementary file 9 — Reporting Summary [file 41467_2019_9303_MOESM9_ESM.pdf]

nature research

Corresponding author(s): Marco Ventura

Reporting Summary

Nature Research wishes to improve the reproducibility of the work that we publish. This form provides structure for consistency and transparency in reporting. For further information on Nature Research policies, see Authors & Referees and the Editorial Policy Checklist.

Statistical parameters

When statistical analyses are reported, confirm that the following items are present in the relevant location (e.g. figure legend, table legend, main text, or Methods section).

n/a	Confirmed	
☐	☒	The exact sample size (n) for each experimental group/condition, given as a discrete number and unit of measurement
☐	☒	An indication of whether measurements were taken from distinct samples or whether the same sample was measured repeatedly
☐	☒	The statistical test(s) used AND whether they are one- or two-sided<br>*Only common tests should be described solely by name; describe more complex techniques in the Methods section.*
☐	☒	A description of all covariates tested
☐	☒	A description of any assumptions or corrections, such as tests of normality and adjustment for multiple comparisons
☐	☒	A full description of the statistics including central tendency (e.g. means) or other basic estimates (e.g. regression coefficient) AND variation (e.g. standard deviation) or associated estimates of uncertainty (e.g. confidence intervals)
☐	☒	For null hypothesis testing, the test statistic (e.g. F, t, r) with confidence intervals, effect sizes, degrees of freedom and P value noted<br>*Give P values as exact values whenever suitable.*
☐	☒	For Bayesian analysis, information on the choice of priors and Markov chain Monte Carlo settings
☐	☒	For hierarchical and complex designs, identification of the appropriate level for tests and full reporting of outcomes
☐	☒	Estimates of effect sizes (e.g. Cohen's d, Pearson's r), indicating how they were calculated
☐	☒	Clearly defined error bars<br>*State explicitly what error bars represent (e.g. SD, SE, CI)*

Our web collection on statistics for biologists may be useful.

Software and code

Policy information about availability of computer code

Data collection	No software were used.
Data analysis	Qiime2-2018.8 for 16S rRNA gene microbial profiling analysis, METAnnotatorX (Milani et al., 2018, Microbiome) for genomics and metagenomics analyses, metaSNV (Costea et al., 2017, PLoS One) for metagenomics analyses and IBM SPSS Statistics for Windows, Version 22.0 for statistics.

For manuscripts utilizing custom algorithms or software that are central to the research but not yet described in published literature, software must be made available to editors/reviewers upon request. We strongly encourage code deposition in a community repository (e.g. GitHub). See the Nature Research guidelines for submitting code & software for further information.

Data

Policy information about availability of data

All manuscripts must include a data availability statement. This statement should provide the following information, where applicable:

- Accession codes, unique identifiers, or web links for publicly available datasets
- A list of figures that have associated raw data
- A description of any restrictions on data availability

Raw sequences of 16S rRNA gene profiling and bifidobacterial ITS profiling as well as shotgun metagenomics sequences are accessible through SRA study accession

number SRP155009 and SRP167296. B. mongoliense BMON18 genomes was deposited under accession number QRAJ00000000. The source data underlying Figures 1, 2, 3, 4 and 5 as well as Supplementary Figures S1, S2, S3, S4 and S5 are provided as a Source Data file.

Field-specific reporting

Please select the best fit for your research. If you are not sure, read the appropriate sections before making your selection.

☐ Life sciences ☐ Behavioural & social sciences ☒ Ecological, evolutionary & environmental sciences

For a reference copy of the document with all sections, see nature.com/authors/policies/ReportingSummary-flat.pdf

Ecological, evolutionary & environmental sciences study design

All studies must disclose on these points even when the disclosure is negative.

Study description	We evaluated the transmission of bacteria across the cheese production chain.
Research sample	We investigated the microbiota composition of a total of 168 samples including stool, litter and milk samples of 50 cows from different husbandries as well as samples of fresh Parmesan cheeses manufactured from the same stocks of milk.
Sampling strategy	Randomized sampling of stool, litter, milk and cheese samples from five cheese making sites.
Data collection	For each cow, fecal samples were collected immediately after defecation, while corresponding milk samples were taken directly by hand during evening milking, after the teat-ends were cleaned and disinfected. Moreover, 10 environmental samples were recovered from litters, while three fresh Parmesan Cheese samples were collected by trimming the fresh rind of the Parmesan cheese shapes produced with the sampled milks, for each husbandry. All samples were kept on ice, shipped under sub-zero conditions to the laboratory and stored at -80 °C until further processing.
Timing and spatial scale	Sampling was performed in the same day for each cheese making site.
Data exclusions	No data were excluded.
Reproducibility	Transmission of bacteria across the cheese production chain was verified for five cheese making sites.
Randomization	Samples were grouped by matrix.
Blinding	Bovine samples from the same cheese making site live together and shared the same foods, water and litters, thus inducing extensive cross-contamination. For this reason, no blinding was performed.

Did the study involve field work? ☐ Yes ☒ No

Reporting for specific materials, systems and methods

Materials & experimental systems

n/a	Involved in the study
☒	☐ Unique biological materials
☒	☐ Antibodies
☒	☐ Eukaryotic cell lines
☒	☐ Palaeontology
☐	☒ Animals and other organisms
☐	☒ Human research participants

Methods

n/a	Involved in the study
☒	☐ ChIP-seq
☒	☐ Flow cytometry
☒	☐ MRI-based neuroimaging

Animals and other organisms

Policy information about studies involving animals; ARRIVE guidelines recommended for reporting animal research

Laboratory animals	n/a
Wild animals	n/a
Field-collected samples	We sampled cows from five cheese making sites located in Parma and Reggio Emilia.

Human research participants

Policy information about studies involving human research participants

Population characteristics	For the pilot study, we enrolled 20 random healthy adults living in Parma and Reggio Emilia.
Recruitment	Recruitment was random among individuals that ate Parmesan cheese (and not other dairy products) daily.
